# Classifying Ten Types of Major Cancers Based on Reverse Phase Protein Array Profiles

**DOI:** 10.1371/journal.pone.0123147

**Published:** 2015-03-30

**Authors:** Pei-Wei Zhang, Lei Chen, Tao Huang, Ning Zhang, Xiang-Yin Kong, Yu-Dong Cai

**Affiliations:** 1 College of Life Science, Shanghai University, Shanghai, P.R. China; 2 The Key Laboratory of Stem Cell Biology, Institute of Health Sciences, Shanghai Institutes for Biological Sciences, Chinese Academy of Sciences, Shanghai, P.R. China; 3 Department of Biomedical Engineering, Tianjin Key Lab of BME Measurement, Tianjin University, Tianjin, P.R. China; 4 College of Information Engineering, Shanghai Maritime University, Shanghai, P.R. China; University of Alberta, CANADA

## Abstract

Gathering vast data sets of cancer genomes requires more efficient and autonomous procedures to classify cancer types and to discover a few essential genes to distinguish different cancers. Because protein expression is more stable than gene expression, we chose reverse phase protein array (RPPA) data, a powerful and robust antibody-based high-throughput approach for targeted proteomics, to perform our research. In this study, we proposed a computational framework to classify the patient samples into ten major cancer types based on the RPPA data using the SMO (Sequential minimal optimization) method. A careful feature selection procedure was employed to select 23 important proteins from the total of 187 proteins by mRMR (minimum Redundancy Maximum Relevance Feature Selection) and IFS (Incremental Feature Selection) on the training set. By using the 23 proteins, we successfully classified the ten cancer types with an MCC (Matthews Correlation Coefficient) of 0.904 on the training set, evaluated by 10-fold cross-validation, and an MCC of 0.936 on an independent test set. Further analysis of these 23 proteins was performed. Most of these proteins can present the hallmarks of cancer; Chk2, for example, plays an important role in the proliferation of cancer cells. Our analysis of these 23 proteins lends credence to the importance of these genes as indicators of cancer classification. We also believe our methods and findings may shed light on the discoveries of specific biomarkers of different types of cancers.

## Introduction

Identifying cancer-specific genes involved in tumorigenesis and cancer progression is one of the major ways to understand the pathophysiologic mechanisms of cancers and to find therapeutic drug targets. Many efforts have been made to identify cancer biomarkers by using gene expression profiles [1]. However, the robustness of microarray-derived biomarkers is very poor [2]; this is in part because the robustness can be easily influenced in gene expression levels by small environmental changes. Without the evaluation of protein expression levels, there would be no way to illustrate causes of tumor proliferation and differentiation. Therefore, better understanding of the translational states of these genomes will bring us a step closer to finding potential drug targets and to illustrating off-target effects in cancer medicine.

Reverse phase protein array (RPPA) is a powerful and robust antibody-based high-throughput approach for targeted proteomics that allows us to quantitatively assess target protein expression in large sample sets [3]. In this process, sample analytes are immobilized in the solid phase, and analyte-specific antibodies are used in the solution phase. Through using secondary tagging and signal amplification to detect bound antibodies, proteins may be measured. Compared with conventional protein quantify methods, such as western blotting or ELISA, the advantages of RPPA include: large-scale quantification of the protein, high sensitivity, and small sample volume requirements [4]. While mass spectrometry, usually used to quantify the numbers of phosphorylation sites or phosphopeptides, requires further protein digestion, peptide fractionation and phosphopeptide enrichment after protein extraction, RPPA can directly quantify the extracted protein [5]. The application of RPPA has been extensively validated for both cell lines and patient samples [6], and it illustrates mechanistic insights behind diseases.

Currently, cancer types are classified by anatomical positions where they are found, such as lung cancer, breast cancer, etc. Whether these names could present their proteomic feature has not been determined until now. Although there have been some methods to find biomarker signatures for specific cancer types, there is still little research being done that considers different types of cancer as a whole in order to identify their similar or distinct proteomic expression patterns and classification features.

In this study, we proposed a computational workflow to successfully use 23 proteins to classify patient samples into ten main cancer types. First, we randomly divided the 3467 samples from ten types of cancers into a training set with 2775 samples and an independent test set with 692 samples. The proportions of each cancer type were similar in the training set and the independent test set. Then, with the training set, all features for distinguishing groups were ranked by the mRMR (minimum Redundancy Maximum Relevance Feature Selection) criteria. With 10-fold cross-validation on the training set, the SMO (Sequential minimal optimization) and the IFS (Incremental Feature Selection) [7] methods were used to choose an optimal feature set. A total of 23 proteins were selected from the training set. Their MCC (Matthews Correlation Coefficient) for the training set was 0.904 evaluated by 10-fold cross validation and their MCC on the independent test set was 0.936. Our methods could provide clinicians with knowledge of key distinct biochemical features of cancer types and could shed some new light on the discoveries of specific biomarkers of different types of cancers.

## Materials and Methods

### Datasets

The RPPA data were downloaded from TCPA (The Cancer Proteome Atlas) database [8] (http://app1.bioinformatics.mdanderson.org/tcpa/_design/basic/download.html under Pan-Cancer 11 RBN), which contained proteomic expression of 3467 cancer patients in 11 cancer types (**Table 1**). Because COAD (Colon adenocarcinoma) and READ (Rectum adenocarcinoma) share similar pathologies and were analyzed together in the TCGA (The Cancer Genome Atlas) colon and rectal cancer study [9], we combined the COAD and READ samples together as 'Colon adenocarcinoma and Rectum adenocarcinoma' samples. Therefore, ten cancer types were analyzed in following steps.

**Table 1:** The ten types of cancers and their sample sizes.

Cancer Type	Cancer Abbreviation	Cancer Name	Sample size	Number of training samples	Number of test samples
1	BLCA	Bladder Urothelial Carcinoma	127	102	25
2	BRCA	Breast invasive carcinoma	747	598	149
3	COAD/READ	Colon adenocarcinoma and Rectum adenocarcinoma	464	371	93
4	GBM	Glioblastoma multiforme	215	172	43
5	HNSC	Head and Neck squamous cell carcinoma	212	170	42
6	KIRC	Kidney renal clear cell carcinoma	454	363	91
7	LUAD	Lung adenocarcinoma	237	190	47
8	LUSC	Lung squamous cell carcinoma	195	156	39
9	OV	Ovarian serous cystadenocarcinoma	412	330	82
10	UCEC	Uterine Corpus Endometrioid Carcinoma	404	323	81
Total			3467	2775	692

Because we did not have a different cohort to do multi-center validation, we randomly divided the 3467 samples into a training set with 2775 samples and an independent test set with 692 samples. The ratio of training samples over test samples was approximately 4:1 and we kept the proportion of each cancer type roughly the same in the training set and the independent test set. The description of the ten cancer types and their sample sizes in are given in **Table 1**. The training and test data sets are provided in **S1 File**.

Each sample contained 187 proteins whose expression levels were measured with reverse phase protein array (RPPA). RPPA is a protein array that allows measurement of protein expression levels in a large number of samples simultaneously in a quantitative manner when high-quality antibodies are available [4]. The 187 protein expression levels were considered as 187 features to be used for the cancer type classifications in this study.

### Feature selection

The expression levels of 187 proteins may not all contribute equally to the classification. The maximum relevance minimum redundancy (mRMR) method [10–13] was employed to rank the importance of the 187 features in the training set. The 187 features can be ordered by using this method according to each feature’s relevance to the target and according to the redundancy among the features themselves.

Let Ω denotes the whole set of 187 features, while Ω_*s*_ denotes the already-selected feature set which includes m features and Ω_*t*_ denotes the to-be-selected feature set which includes n features. The relevance *D* of the feature *f* in Ω_*t*_ with the cancer classes *c* can be calculated by:

D=I(f,c) (1)

And the redundancy *R* of the feature *f* in Ω_*t*_ with the already-selected features in Ω_*s*_ can be calculated by:

$$R=\frac{1}{m}\sum_{f_{i}\in \Omega_{s}}I(f,f_{i})$$ (2)

To obtain the feature *f*_*j*_ in Ω_*t*_ with maximum relevance with cancer classes *c* and minimum redundancy with the already-selected features Ω_*s*_, Equation (1) and Equation (2) are combined as the mRMR function:

$$\underset{f_{j}\in \Omega_{t}}{\max}\left[I(f_{j},c)-\frac{1}{m}\sum_{f_{i}\in \Omega_{s}}I(f_{j,}f_{i})\right](j=1,2,...,n)$$ (3)

The feature evaluation will continue 187 rounds. After these evaluations, a ranked feature list *S* by mRMR method can be obtained:

$$S=\left\{f_{1}{}^{'},f_{2}{}^{'},...,f_{h}{}^{'},...,f_{N}{}^{'}\right\}$$ (4)

The feature index h indicates the importance of feature. A feature with a smaller index h indicated that it had a better trade-off between the maximum relevance and the minimum redundancy, and it may contribute more in the classification.

Based on the ranked feature list in the mRMR table, we adopted the Incremental Feature Selection (IFS) method [14, 15] to determine the optimal feature set, or one that achieves the best classification performance. To perform this method, features in the mRMR table were added one by one from higher to lower rank.

When another feature had been added, a new feature set was generated. And we get 187 feature sets, and the i-th feature set is:

$$S_{i}=\{f_{1},f_{2},...,f_{i}\}\;(1\leq i\leq N)$$ (5)

Based on each of the 187 feature sets, the classifiers were built and tested on the training set with 10-fold cross validation. With Matthews Correlation Coefficient (MCC) of 10-fold cross validation calculated on training set, we obtain an IFS table with the number of features and the performance of them. *S*_optimal_ is the optimal feature set that achieves the highest MCC on training set. At last, the model was build with features from *S*_optimal_ on training set and elevated on the test set.

### Prediction methods

**Fig 1:**
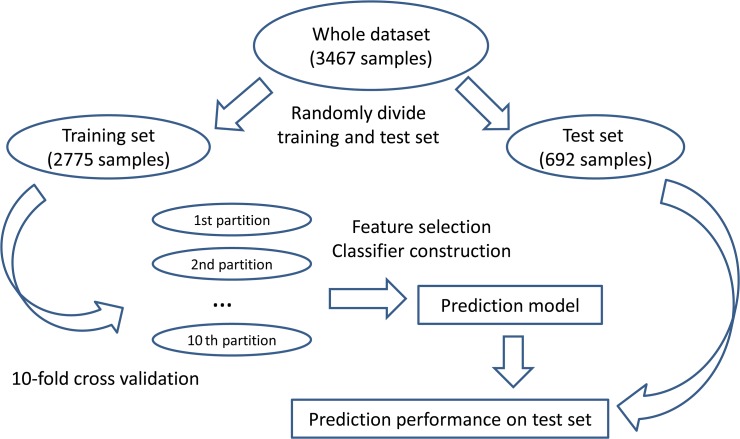
The workflow of model construction and evaluation. First, we randomly divided the whole data set into a training set and an independent test set. Then, the training set was further partitioned into 10 equally sized partitions to perform 10-fold cross validation. Based on the training set, the features were selected and the prediction model was built. At last, the constructed prediction model was tested on the independent test set.

We randomly divided the whole data set into a training set and an independent test set. The training set was further partitioned into 10 equally sized partitions. The 10-fold cross-validation on the training set was applied to select the features and build the prediction model. The constructed prediction model was tested on the independent test set. The framework of model construction and evaluation was shown in **Fig 1**.

We tried the following four machine learning algorithms: SMO (Sequential minimal optimization), IB1 (Nearest Neighbor Algorithm), Dagging, RandomForest (Random Forest), and selected the optimal one to construct the classifier. The brief description of these algorithms was as below.

The SMO method is one of the popular algorithms for training support vector machines (SVM) [16]. It breaks the optimization problem of a SVM into a series of the smallest possible sub-problems, which are then solved analytically [16]. To tackle multi-class problems, pairwise coupling [17] is applied to build the multi-class classifier.

IB1 is a nearest neighbor classifier, in which the normalized Euclidean distance is used to measure the distance of two samples. For a query test sample, the class of a training sample with minimum distance is assigned to the test sample as the predicted result. For more information, please refer to Aha and Kibler’s study [18].

Dagging is a meta classifier that combines multiple models derived from a single learning algorithm using disjoint samples from the training dataset and integrates the results of these models by majority voting [19]. Suppose there is a training dataset ℑ containing *n* samples. *k* subsets are constructed by randomly taking samples in ℑ without replacement such that each of them contain *n*′ samples, where *kn*′ ≤ *n*. A selected basic learning algorithm is trained on these *k* subsets, thereby inducing *k* classification models *M*_1_,*M*_2_,…,*M*_*k*_. For a query sample, *M*_*i*_(1≤*i*≤*k*) provides a predict result and the final predicted result of Dagging is the class with most votes.

Random Forest algorithm was first proposed by Loe Breiman [20]. It is an ensemble predictor consisting of multiply decision trees. Suppose there are *n* samples in the training set and each sample was represented by *M* features. Each tree is constructed by randomly selecting *N*, with replacement, from the training set. At each node, randomly select *m* features and select the optimized split to grow the tree. After constructing multiply decision trees, the predicted result of a given sample is the class that receives the most votes from these trees.

### Matthews Correlation Coefficient (MCC)

MCC [21], a balanced measure even if the classes are of very different sizes, is often used to evaluate the performance of prediction methods on a two-class classification problem. To calculate the MCC, one must count four values: true positives (TP), false positive (FP), true negative (TN) and false negative (FN) [22, 23]. Then, the MCC can be computed by

$$\text{MCC}=\frac{TP\cdot TN-FP\cdot FN}{\sqrt{\left(TN+FN)\cdot (TN+FP)\cdot (TP+FN)\cdot (TP+FP\right)}}$$ (6)

However, many problems involve more than two classes, say *N* classes encoded by 1,2,…,*N* (*N* > 2). In this case, we can calculate the MCC for class *i* to partly measure the performance of prediction methods by counting TP, FP, TN and FN as following manners:

TP_*i*_: the number of samples such that class *i* is their predicted class and true class;

FP_*i*_: the number of samples such that class *i* is their predicted class and class *i* is not their true class;

TN_*i*_: the number of samples such that class *i* is neither their predicted class nor their true class;

FN_*i*_: the number of samples such that class *i* is not their predicted class and class *i* is their true class.

Accordingly, MCC for class *i*, denoted by MCC_*i*_, can be computed by

$${\text{MCC}}_{i}=\frac{TP_{i}\cdot TN_{i}-FP_{i}\cdot FN_{i}}{\sqrt{\left(TN_{i}+FN_{i})\cdot (TN_{i}+FP_{i})\cdot (TP_{i}+FN_{i})\cdot (TP_{i}+FP_{i}\right)}}$$ (7)

However, these values can’t completely measure the performance of prediction methods, the overall MCC in multiclass case is still necessary. Fortunately, Gorodkin [24] has reported the MCC in multiclass case, which was used to evaluate the performance of the prediction methods mentioned in Section “Prediction methods”. In parallel, The MCC for each class will also be given as references. Here, we gave the brief description of the overall MCC in multiclass case as below.

Suppose there is a classification problem on *n* samples, say *s*_1_,*s*_2_,…,*s*_*n*_, and *N* classes encoded by 1,2,…,*N*. Define a matrix *Y* with *n* rows and *N* columns, where *Y*_*ij*_ = 1 if the *i*-th sample belongs to class *j* and *Y*_*ij*_ = 0 otherwise. For a classification model, its predicted results on the problem can be represented by two matrices *X* and *C*, where *X* has *n* rows and *N* columns,

$$X_{ij}=\{\begin{array}{ll} 1 & if\text{ the }i\text{-th sample is predicted to be class }j\\ 0 & \text{otherwise} \end{array}$$ (8)

and *C* has *N* rows and *N* columns, *C*_*ij*_ is the number of samples in class *i* that have been predicted to be class *j*.

For Matrices *X* and *Y*, their covariance function can be calculated by

$$\text{cov}(X,Y)=\frac{1}{N}\sum_{k=1}^{N}\text{cov}(X_{k},Y_{k})=\frac{1}{N}\sum_{i=1}^{n}\sum_{k=1}^{N}\left(X_{ik}-{\bar{X}}_{k})(Y_{ik}-{\bar{Y}}_{k}\right)$$ (9)

where *X*_*k*_ and *Y*_*k*_ are the *k*-th column of matrices *X* and *Y*, respectively, ${\bar{X}}_{k}$ and ${\bar{Y}}_{k}$ are mean value of numbers in *X*_*k*_ and *Y*_*k*_, respectively. Then, the MCC in multiclass case can be computed by the following formulation [25]:

$$MCC=\frac{\text{cov}(X,Y)}{\sqrt{\text{cov}(X,X)\text{cov}(Y,Y)}}=\frac{\sum_{k,l,m}^{N}\left(C_{kk}C_{ml}-C_{lk}C_{km}\right)}{\sqrt{\sum_{k=1}^{N}\left[(\sum_{l=1}^{N}C_{lk})(\sum_{f,g=1,f\neq g}^{N}C_{gf})\right]}\sqrt{\sum_{k=1}^{N}\left[(\sum_{l=1}^{N}C_{kl})(\sum_{f,g=1,f\neq g}^{N}C_{fg})\right]}}$$ (10)

Like the MCC in two-class case, the MCC in multiclass case ranges between -1 and 1, where 1 indicates the perfect classification, -1 the extreme misclassification.

## Results and Discussion

### The mRMR and IFS results

By using the maximum relevance minimum redundancy (mRMR) method, the 187 features were ranked by importance in the training set. The result of the mRMR table can be found in **S2 File**.

**Fig 2:**
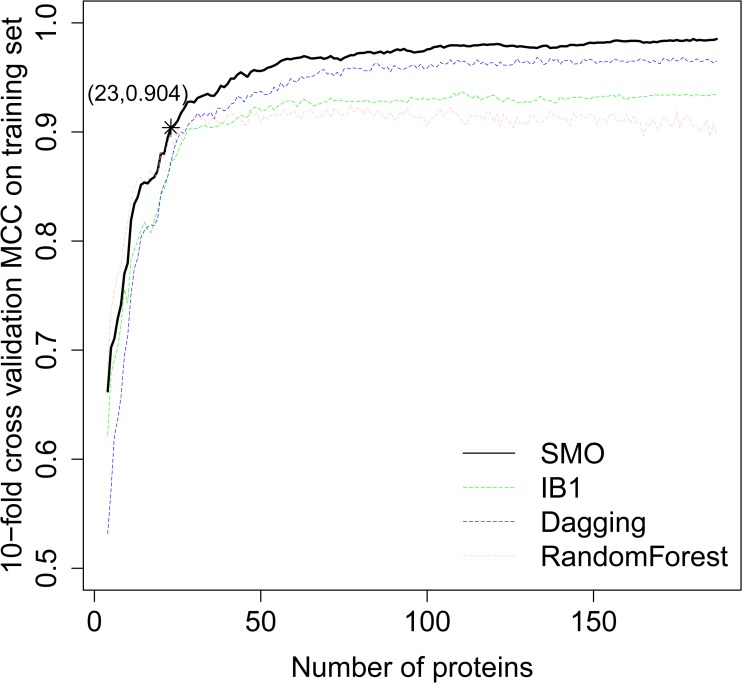
The IFS curves for the classifying of the 10 types of tumors. Plot to show the MCCs of the different classifiers constructed by different number of protein features selected from the mRMR table during the IFS process on training set. When the first 23 proteins were selected, the MCC reached 0.904, which was the first reach above 0.900 and with more protein features, the MCC did not increase much. We considered the 23 proteins as the most significant proteins for the classification.

During the IFS approach, each protein feature was added one by one. The classification MCCs which were obtained by four prediction methods, on the training set evaluated by 10-fold cross validation are presented in **S3 File**. We depicted the classification MCCs as **Fig 2**from the data in **S3 File**. It can be observed that the highest MCCs for SMO, IB1, Dagging and RandomForest were 0.985, 0.937, 0.969 and 0.925, indicating SMO can be used to construct an optimal classifier. By carefully checking the predicted results of SMO, it can be seen that by using the top 23 proteins, the MCC reached 0.904 which was the first reach above 0.900. With more proteins, the MCC did not increase by much. Therefore, in this study, we considered the 23 proteins as the optimal feature set and these 23 proteins were regarded as the most important proteins in classifying these ten types of cancers. We evaluated their prediction performance on the independent test set and the MCC was 0.936. The MCC for each cancer type can be found in **S3 File**.

### The selected top 23 proteins for distinguishing cancer types

**Fig 3:**
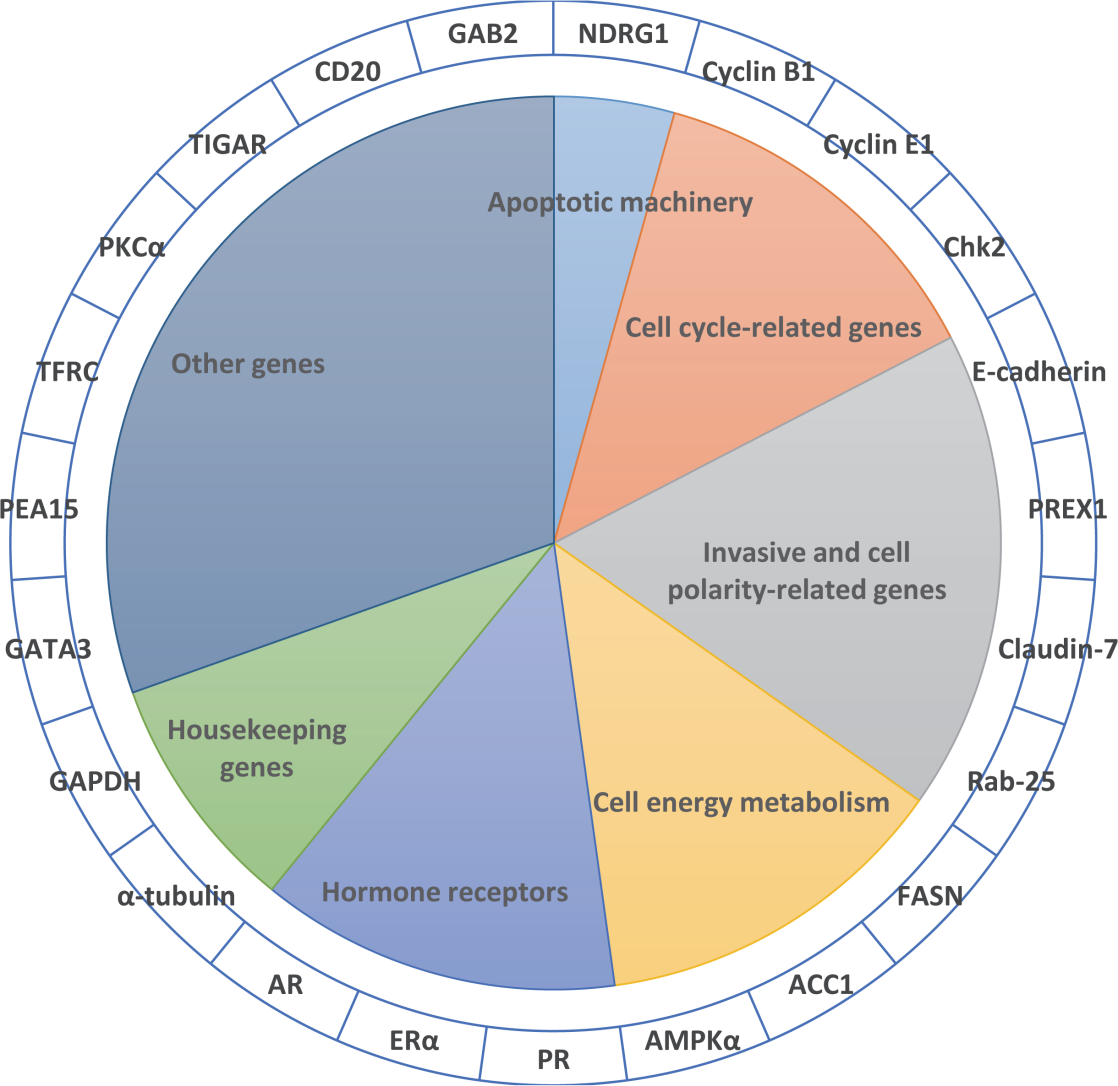
The 23 selected proteins. The 23 selected proteins are ascribed to seven sections mainly based on hallmarks of cancer. For those that are not associated with cancer-related pathways, we put genes with similar functions together to discuss.

The selected top 23 proteins are summarized in **Table 2**. These proteins may play important roles in classifying the ten different cancer types. Most of these proteins have been reported to be related to certain tumors. For example, Claudin-7 has been reported to be over-expressed in breast tumors [26] and down-regulated in head and neck carcinomas [27]. TIGAR is up-regulated in colon tumors [28]. Gene amplification of ESR1 occurs frequently with breast cancer [29]. PREX1 is highly expressed in prostate cancer [30]. Thus, our findings are further corroborated by these previous results. Below, we will discuss the biological significance of the 23 proteins in detail based on gene function, cell pathways and biological functions, which may shed some light on the differences of different cancers in protein expression levels. We mainly discuss these genes in sections according to Robert A. Weinburg’s [31]. For some genes that do not apply to cancer’s hallmarks, we try to put these genes with similar functions together for discussion (see **Fig 3**).

**Table 2:** The top 23 important proteins for the classification of the 10 cancer types.

Order	Name	Gene Name	Protein function and regulatory pathways
1	FASN	FASN	Fatty acid synthase (FASN) catalyzes the synthesis of long-chain fatty acids from acetyl-CoA and malonyl-CoA. Indicated as a poor prognosis in breast and prostate cancer.
2	Claudin-7	CLDN7	Claudins make up tight junction strands.
3	PR	PGR	Progesterone receptor, Transcription Factor
4	TIGAR	C12ORF5	Regulates p53 tumor suppressor pathway and glycolysis
5	GATA3	GATA3	Transcription factor
6	NDRG1_pT346	NDRG1	A member of the NDRG family functions in growth, differentiation, and cell survival
7	AR	AR	Androgen receptor (AR). Transcription Factor
8	PREX1	REX1	Downstream of Heterotrimeric G proteins (Guanine nucleotide exchange factor)
9	PEA15_pS116	PEA15	Implicated in the regulation of multiple cellular processes including apoptosis, integrin activation, and insulin-sensitive glucose transport in insulin-responsive cells. Its activation is mediated through binding to multiple proteins, including ERK1&2, RSK2, Akt, FADD, and Caspase-8.
10	Cyclin_B1	CCNB1	Cyclin B1 regulates mitosis. Cyclin B1 levels rise during S phase and G2, and peak at mitosis.
11	ER-alpha	ESR1	Estrogen receptor, Transcription Factor
12	AMPK_alpha	PRKAA1	Involved in energy homeostasis regulation
13	Acetyl-a-Tubulin-Lys40		The cytoskeleton consists of three types of cytosolic fibers: microtubules, microfilaments (actin filaments), and intermediate filaments. Acetylation of α-tubulin at Lys40 is required for dynamic cell shape remodeling, cell motility, tubulin stability and terminal branching of cortical neurons
14	Rab-25	Rab-25	A member of Rab11 family possesses small Ras-like GTPase activity. Increased Rab25 expression is associated with aggressive growth in ovarian and breast cancer, where Rab25 may inhibit apoptosis and promote cancer cell proliferation and invasion through regulation of vesicle transport and cellular motility.
15	Chk2	CHEK2	Kinase acts downstream of ATM/ATR involving in DNA damage checkpoint control, embryonic development, and tumor suppression
16	E-Cadherin	CDH1	A member of transmembrane glycoprotein superfamily, Mediate calcium-dependent cell-cell adhesion and normal tissue development.
17	ACC1	ACACA	Key enzyme in the biosynthesis and oxidation of fatty acids. Involved in energy homeostasis regulation
18	GAPDH	GAPDH	Glyceraldehyde 3-phosphate dehydrogenase
19	PKC-alpha_pS657	PRKCA	PKC alpha is an ubiquitously expressed PKC isozyme that has been implicated in the regulation of a broad range of cellular functions
20	TRFC	TFRC	Transferrin Receptor
21	Cyclin_E1	CCNE1	Cyclin E has been found to be associated with the transcription factor E2F in a temporally regulated manner. The cyclin E/E2F complex is detected primarily during the G1 phase of the cell cycle and decreases as cells enter S phase. E2F is known to be a critical transcription factor for expression of several S phase specific proteins.
22	CD20	CD20	A surface molecule of B-lymphocyte during the differentiation of B-cells into plasma cells
23	GAB2	GAB2	A docking protein, which mainly mediates the interaction between receptor tyrosine kinases (RTKs) and non-RTK receptors.

Preventing cell death is crucial for cancer development because cancer cells are often resistant to apoptotic signaling caused by DNA damage and other factors. In our results, we found one gene that is related to apoptotic machinery and could be used to distinguish different cancers. Here, we discuss NDRG1, as well as previous findings showing its relationship to cancer. NDRG1 (N-myc downstream regulated gene 1) is a phosphorylated protein [32] that could be activated by the tumor suppressor gene p53 and required for the induction of p53-mediated apoptosis in the colon cancer cell line [33]. Because the NDRG1 protein has a crucial role in inhibiting primary tumor growth, it is well-known as a metastasis suppressor in a number of cancers including colon, prostate and breast cancers [34].

Replicative immortality is an important hallmark of cancer, which is commonly recognized as deregulated cell proliferation. Our findings on several important cell cycle-related genes in selected proteins not only illustrate their importance to the development of cancer, but are also first used as indicators of cancer classification. These cell cycle-related genes are discussed below: Cyclin B1 has a role in the regulation of cell cycle: before entering mitosis, cells flip between G2 and mitosis until there is sufficient accumulation of cyclin B to support CDK1 activity [35]. Misexpressed cyclin B1 in the nucleus has been found in a huge proportion of cells of some neoplasms, and cyclin B1 has been regarded as a potent prognostic factor in human breast carcinoma and squamous cell carcinoma [36]. Cyclin E1, encoded by CCNE1, is one of the members of the cyclin family, which controls cell cycle processes by dramatic periodicity of abundance. Recently, a genome-wide association study found that rs8102137 within the CCNE1 gene is associated with bladder cancer [37]. Meta-analysis also indicates that there is over-expression of this protein with breast cancer [38]. Chk2 (checkpoint kinase 2), as a serine/ threonine protein kinase, could respond to DNA damage in order to maintain genomic integrity [39]. It has been shown that Chk2 plays an important role in the proliferation of cancer cells [40], attracting much attention to make it a possible anti-cancer drug design target [41].

It is clear that invasion is a hallmark of cancer, even if its underlying mechanisms are still an enigma. Until now, the gain and loss of cell-cell attachment proteins are the main reasons of invasion, especially the loss of E-cadherin [31]. In our results, E-cadherin and some polarity-related proteins are found that could be used to distinguish different cancer types. These proteins are discussed below: E-Cadherin, as the type-1 classical cadherin, mediates cell interactions. Tumor progression is often linked with the loss of E-cadherin function, leading to a more motile and invasive phenotype [42]. PREX1 (phosphatidylinositol-3,4,5-trisphosphate-dependent Rac exchange factor) is highly expressed in prostate cancer, indicating a relationship between the cell invasion and its expression [30]. In melanomas, PREX1 over-expression was connected to the activation of ERK-MAPK signaling and required for efficient melanoblast metastasis as well as for migration [43]. Claudin-7, a common transmembrane protein, plays a vital role in the formation and maintenance of the permeability in polarized epithelial cells [44]. The aberrant Claudin-7 expression profile has been found in various tumors, such as highly induced Claudin-7 expression in both primary and metastatic breast tumors, [26] yet it is down-regulated in head and neck carcinomas [27]. These previous studies further supported our findings that Claudin-7 could be used as a biomarker for the differentiation and classification of various tumors. Rab-25, as a member of the Rab family of GTPases, Rab-25 is a constitutively active Rab GTPase that plays a crucial role in apical recycling and transcytosis pathways in polarized epithelial cells. Because loss of cell polarity is an essential hallmark of cancer, Rab-25 related trafficking has an important impact on epithelial cell polarity program in cancer progression [45].

Anomalous cancer cell energy metabolism was first observed by Otto Warbugy in 1930 and has been accepted as a hallmark of cancer. Abnormal fatty-acid synthesis as one type of energy metabolism is found in many cancer cells [46]. Here, several important fatty acid and glycolytic metabolism-related genes are found in the selected 23 proteins: FASN is a key enzyme which is required for de novo synthesis of fatty acid. It has been found that the FASN expression and activity are abnormally elevated in many types of human cancers, which may contribute to cellular resistance to drug- and radiation-induced apoptosis [46]. ACC1 is a rate-limiting enzyme in de novo fatty acids synthesis. It seems to be the limiting enzyme in proliferating cancer cells. ACC1 has been found to be up-regulated in proliferating cancer cell lines such as prostate, breast and liver. Indeed, it has been shown that knock-down of ACC1 by siRNA promotes apoptosis in prostate cancer and breast tumor cells but not in control noncancerous cells, underlining cancer cells' higher reliance on this enzyme than normal tissue [47]. AMPK (AMP-activated protein kinase, encoded by the gene PRKAA1/2) plays a crucial role in sensing available energy and coordinating external growth signals with cellular metabolism [48]. A decrease of AMPK signaling, mostly caused by the loss of function gene STK11, could lead to increased activation of mTOR and a shift toward glycolytic metabolism, which is found in a variety of cancers, including NSCLC [49] and cervical cancer [50].

Abnormal expression of hormone receptors are often shown in sex-related cancers, such as breast cancer and prostate cancer. Three hormone receptors are also reported in the selected proteins: Progestin receptor (PR), as a nuclear steroid receptor, has a high specificity for binding progesterone [51]. It has been shown in literature that PR inhibits the transition from G1 to S in the cell cycle and promote apoptosis in endometrial cancer cells [52]. In the GOG119 phase II trial, an estrogen surrogate named tamoxifen could enhance progestin activity in order to induce PR and cure endometrial patients [53]. Estrogen receptor (ER, activated by the hormone estrogen) is one of the most important therapeutic targets in breast cancers, given that the correlation between ER expression and cellular response to estrogen [54]. It has been reported that gene amplification of ESR1 frequently occur with breast cancer [29]. Androgen receptor (AR; NR3C4) is believed to solely mediate all the biological actions of endogenous, functioning mainly in regulating male development. Due to the strong connection between ARs and prostate cancer, androgen antagonists or androgen deprivation therapy has been applied to impede cancer cell proliferation of patients with androgen-dependent prostate cancer in clinical treatment [55].

Surprisingly, among these 23 selected proteins that are used to distinguish different cancers, α-tubulin and GAPDH are often used as controls in western blot analysis. In the following part, we will discuss known findings about α-tubulin and GAPDH that lend credence to the validity of our findings for their importance to distinguish cancers. For example, both α- and β- tubulin proteins are responsible for assembling microtubules (MTs, cytoskeletal polymeric structures), and certain posttranslational modifications. The acetylation of α-tubulin (Lys-40) [56] could alter dynamic behavior of MTs, which may lead to changes in biological functions that MTs perform during cell division, migration, and intracellular trafficking. Taking the dynamic parameters into account, MTs provide an attractive target for chemotherapy against rapidly growing tumor cells such as in lymphoma and leukemia, metastatic cancers, and slow growing tumors of the breast, ovary, and lung [57, 58]. Over the last decade, GAPDH (glyceraldehyde-3-phosphate dehydrogenase) was considered a housekeeping gene and was as a control for equal loading during the experimental process. However, it has been shown that GAPDH expression varies different types of tissues. Moreover，GAPDH expression varies due to oxygen tension [59], and the expression levels of GAPDH vary in fallopian tube cancers and ovarian cancers [60]. On the basis of GAPDH’s predilection for AU-rich elements, it has been shown that GAPDH can bind to the CSF-1 3'UTR that stabilize the mRNA [60]. To summarize, combining all the evidence, tubulin proteins and GAPDH may bring a new perspective on cancer studies, and it is suggested that they are not used as controls in western blot analysis of different types of cancer.

Other selected proteins include phosphatases, transcriptional activators, linker proteins and transferrin receptors: GATA3 is a transcriptional activator with high expression levels [61] and the third most frequently mutated gene in breast cancer [62]. Thus, GATA3 has proved to be a useful immunohistochemical marker to predict tumor recurrence early in the progression of breast cancer. PEA15, as a multifunctional linker protein predominantly expressed in the cells of the nervous system, such as astrocytes [63], controls a variety of cellular processes, such as cell survival, proliferation, migration and adhesion [64]. PEA15 functions in various cancers, concluding glioblastoma, astrocytoma, and mammary, as well as skin cancers. PEA15 can have both anti- (in ovarian carcinoma [65]) and pro- (glioblastoma [66]) tumorigenic functions, depending on its interactions. TFRC is a transferrin receptor. It is a major iron importer in most mammalian cells. It has been shown that TFRC proteins increase in breast, malignant pancreatic cancer, and other cancers [67, 68]. PKCα is encoded by PRKCA gene and is a serine- and threonine- specific kinase. This gene is highly expressed in multiple cancers, and the high activation of PKCα has been identified to promote the genesis of breast cancer [69]. The high abundance in serum makes this protein to be a good diagnostic biomarker of lung cancer [70] and gastric carcinoma [71]. TIGAR is a fructose-2-6-bisphosphatase that promotes the production of antioxidant (NADPH) and nucleotide synthesis material (ribose-5-phosphate) and seems to be important for tissue renewal and intestinal tumorigenesis. Up-regulated expression of TIGAR in human colon tumors along with other evidence suggest its importance in the development of cancer and metabolism regulation and may be used as a therapeutic target in diseases such as intestinal cancer [28]. CD20 (Membrane-Spanning 4-Domains, Subfamily A, Member 1, MS4A1) encodes a surface molecule B-lymphocyte during the differentiation of B-cells into plasma cells. Currently, a CD20 monoclonal antibody has been utilized in the treatment of cancer, even though its dosage is still under discussion [72]. GAB2 (GRB2-associated-binding protein 2) is a docking protein, which mainly interacts with signaling molecules. Research has shown that the oncogenesis of many cancers including gastric, colon, ovarian and breast cancer is related to GAB2 [73, 74]. For example, GAB2 can amplify the signal of receptor tyrosine kinases (RTKs), which plays roles in breast cancer development and progression [75].

As shown above, all of the top 23 proteins are closely related to certain types of cancers. Researchers have focused on common features of different cancer types for decades [31]. Admittedly, in theory, the hallmarks of cancer would help us develop drugs to treat all types of cancers as a whole. However, this “one size fits all” cancer treatment has disappointed us due to its treatment-related toxicity and inefficiency. Despite the fact that personalized treatments have been proposed, the theory still stays at a conceptual phase. Thus, having a better understanding of the potential values and the applied ranges of cancer drugs based on different biomarkers may be a more realistic way to treat different types of cancers.

### Potential values of our findings

Previous experimental studies in the literature could consolidate our results showing that the selected 23 proteins could be used as biomarkers for certain cancers. They also can explain partially why the combination of these proteins could be used to accurately classify different cancer types. However, to our knowledge, reasons behind the varying expression patterns in different types of cancers have not been found. At least, by using our computational method, one could gain a better understanding of the similarities and differences among different cancers. This could help us identify proteins that could promote the development of cancers and proteins that might not be indispensable for cancer development. Further studies should be performed to determine whether the differential expression patterns of proteins in various cancers are influenced by their original tissues. Those proteins specifically expressed in certain types of cancers could be considered as potential specific cancer targets, which could be used to improve the target efficiency. Therefore, our results may help drug designers obtain a better understanding of the potential targets of drugs by shedding some light on the cancer type-specific biomarker discoveries.

## Supporting Information

S1 File The dataset used in this study. There were 3467 cancer patient samples in 10 cancer types, with 187 proteins for each sample. The 3467 samples were randomly divided into 2775 training samples and 692 independent test samples. The first column is the sample ID, the second column is the cancer types whose description can be found in Table 1. The third to the 189th columns were proteins.
(XLSX)

S2 File The mRMR table. All the 187 protein features were ranked from the most important to the least by using the mRMR method on training set. The top 23 proteins were regarded as composing the optimal feature set because by using the 23 protein features, the MCC on the training set evaluated by 10-fold cross validation reached 0.904 which was the first reach above 0.900, and with more protein features, the MCC did not increase much.
(XLSX)

S3 File The classification MCCs of four prediction methods, SMO (Sequential minimal optimization), IB1 (Nearest Neighbor Algorithm), Dagging and RandomForest (Random Forest), on the training set evaluated by 10-fold cross validation and the MCC of SMO with 23 features on test set. (XLSX)
